# Relative acquisition evaluation of health poverty alleviation project: a quantitative study of data from different regions of China

**DOI:** 10.1186/s12889-022-14703-7

**Published:** 2022-11-28

**Authors:** Xinjie Zhang, Ying Luo, Wei Chen, Jingru Huang

**Affiliations:** 1 Department of Public Administration, School of Management, Jiangsu University, Zhenjiang, China; 2 Department of Personnel and Social Security, School of Governmentment, Nanjing University, Nanjing, China; 3 Administration Office, Affiliated Hospital of Jiangsu University, Zhenjiang, China

**Keywords:** Capability approach, Health poverty alleviation project, Relative acquisition, Equity, Evaluation

## Abstract

**Background:**

Poverty is the greatest obstacle to the realization of human rights, among which illness is the leading cause in China. In 2015, China began to implement the health poverty alleviation project (HPAP). By 2020, all rural households living below the current poverty level (2300 yuan per person per year) have been lifted out of poverty.

**Methods:**

This study introduces the concept of relative acquisition and constructs a scale based on the capability approach to measure the relative acquisition and compares its fairness of HPAP.

**Results:**

The findings show that the values of the relative acquisition of HPAP in survey areas are all reached middle level (0.4–0.6), with 0 indicating the worst level and 1 indicating the best level. Specifically, the values of the functional activities of "health care", "health ability", "equal treatment opportunities" and "social support" are all above 0.4, while the values of "economic conditions" and "health education" are below 0.4.

**Conclusions:**

The HPAP plays a significant role in reducing the economic burden of disease on patients. However, due to insufficient social support and health education, the HPAP objects lack endogenous motivation to fight against poverty, and the fairness also needs to be improved.

## Background

Poverty is the biggest obstacle to the realization of human rights. According to the latest World Bank data, 8.4% of the world's population was still in poverty in 2019, having an average daily income of $1.9 [1]. Among the causes of poverty, illness is the most prominent. Because the effects of illness are damaging and have a long-term impact on the economic conditions of patients and their families. China is no exception to this rule. At the end of 2015, the proportion of poor households with files in China increased to 44.1% due to illness [2]. To achieve the goal of poverty alleviation by 2020, the CPC Central Committee and The State Council issued “the Decision on Winning the Battle against Poverty” in 2015, which proposed the implementation of the health poverty alleviation project (HPAP). This project, led by the government, requires all regions to accurately identify and document poor people based on the national poverty alleviation standard (2300 yuan per person per year in 2010) [3] and to prevent poverty or reoccurrence of poverty caused by disease through the precise implementation of medical assistance and improvement of medical conditions. According to World Bank standards, this alleviation accounted for more than 70% of the change throughout the world in the same period and achieved the goal of the 2030 Agenda for Sustainable Development 10 years ahead of schedule [4].

At the same time, scholars have conducted extensive studies on poverty and its governance. Some studies indirectly evaluated the effect of HPAP from the perspective of specific measures. For example, Wang NS et al. and Zhai SG et al. [5, 6] studied the effect of medical insurance system on reducing the economic burden of disease among the elderly and related vulnerable groups in rural areas. Some scholars have directly analyzed the implementation effects of HPAP [7, 8]. However, only Wei Y et al. [9] paid attention to both objective and subjective indicators in the evaluation of the effect of HPAP. In general, the existing studies are insufficient in the aspects of evaluation perspective, variable selection and data pertinence.

The concept of relative acquisition provides a new evaluation direction for HPAP. Compared to the "happiness", relative acquisition places stronger emphasis on real acquisition [10–12]. However, there is no consensus on the concept of relative acquisition [13–15]. We think that the relative acquisition of HPAP is an actual acquisition and subjective feeling of poverty alleviation objects in the process of being assisted, which is mainly measured by comparison with one’s past self and other individuals in social relationships. And the former is called vertical acquisition (VA) and the latter is called horizontal acquisition (HA).

Although the poverty caused by illness has been alleviated to some extent in recent years, there are still obvious gaps in income level, medical security and service accessibility among different people and regions, which may exacerbate the occurrence of catastrophic expenditure and trigger health inequality [16–19]. The capability approach, put forward by Sen A [20], which offers a possibility to explain and evaluate the relative acquisition of HPAP in the process of development. Sen A believes that substantive freedom is the goal of social development, which should be evaluated by capability. People who are deprived of basic capability will fall into poverty [21]. This theory lays the foundation for the world to understand poverty from the multi-dimensional perspectives of economy, education, health and so on [22–25]. Based on this, this study builds a comprehensive evaluation scale to analyze the effect and equity of HPAP.

The main contributions of this study are aspects: First, the concept of relative acquisition is clarified, which lays a foundation for scale design, data screening and outcome measurement; Second, under the framework of the capability approach, this study comprehensively consider data acquisition, research hypotheses, social consensus and other factors to determine the categories and indicators of functional activities, which increases the scientificity of scale; Third, based on the data from extensive field research, this study measures the relative acquisition in different regions, which not only increases the applicability of its conclusions but also promotes the establishment of a long-term mechanism for poverty alleviation.

## Methods

### Study design

**Fig. 1:**
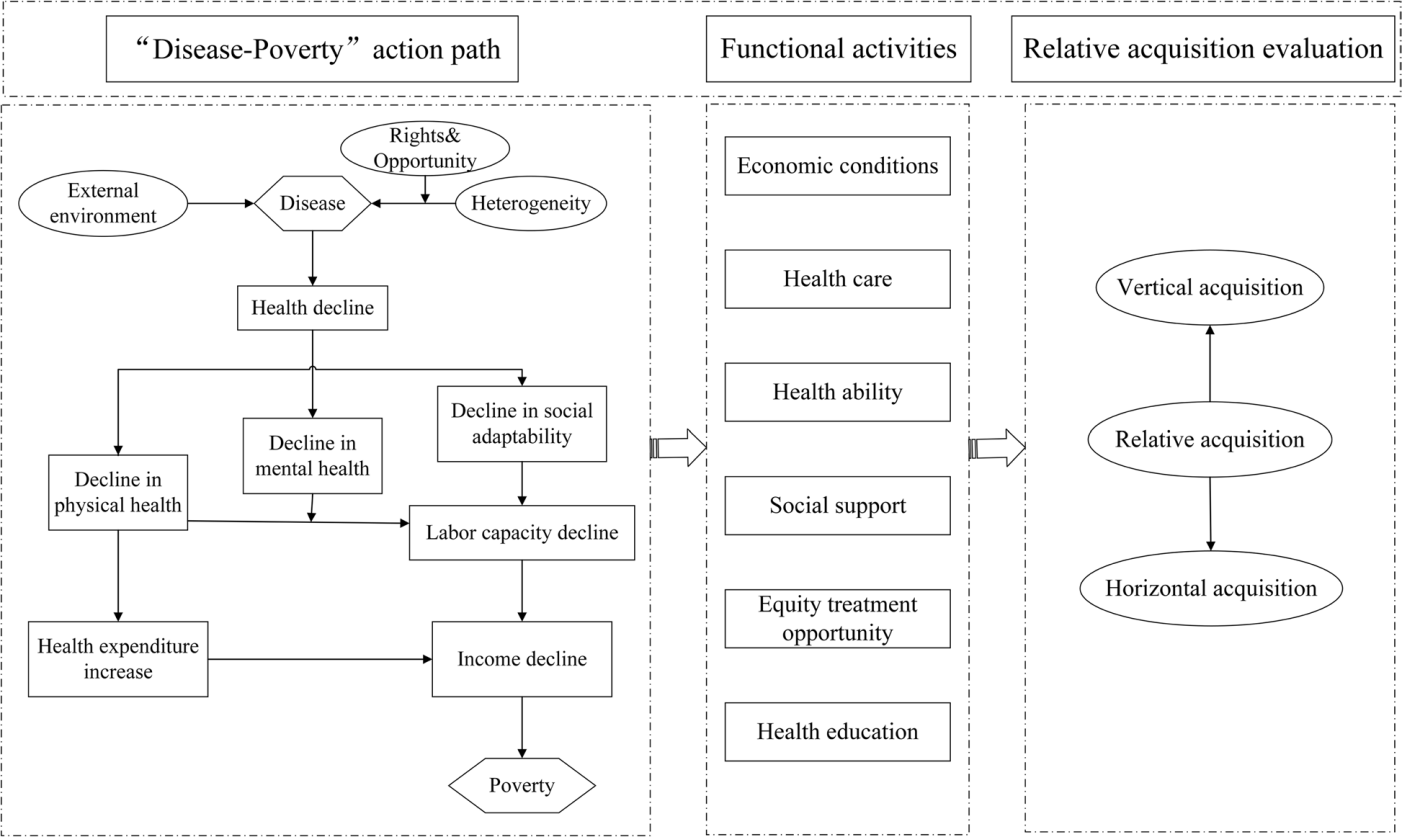
Evaluation model of the relative acquisition of HPAP based on the capability approach

As we all know, the relationship between diseases and poverty is not linear, but overlapping, as shown in Fig. 1. In general, critical illnesses and long-term chronic diseases may lead to poverty, even for people of better social and economic status [6]. The damage of such diseases to patients is often fundamental and may leads to a long-term decline in many patients' capabilities and forms a vicious circle. Although economic assistance can reduce the burden of disease in the short term, but in the long run, individual differences may aggravate illness or even return the patient to poverty due to lack of social support, equal treatment opportunities, health education and so on. The capability approach provides ideas and directions for analyzing this problem. Based on the analysis of the effects of disease on poverty, this study established a theoretical model for evaluating the relative acquisition of HPAP, which mainly includes six functional activities, namely, "economic conditions", "health care", "health ability", "social support", "health education" and "equal treatment opportunities".

At this stage, a total of 13 interviewees were selected, including 10 HPAP objects and 3 front-line workers. The HPAP objects are mainly randomly selected from the range which is determined by survey areas based on national poverty alleviation standards and in combination with local reality. The interview on HPAP objects mainly includes three aspects: first, the status of disease and economic burden; second, the influence of disease on health, life and work; third, the overall evaluation of HPAP on health ability, social support and medical security. And the interview with front-line workers also focused on three aspects: first, measures have been taken to prevent poverty in survey area; second, the main effects of HPAP and the relative acquisition of HPAP objects; and third, the problems and suggestions for HPAP. The entire interview was conducted in a semi-structured manner.

After the interview, two researchers encoded and categorized the content of the interviews using the *Holsti* reliability calculation Formula (1) to test the consistency of coding and the accuracy of classification [26] and finally formed 20 evaluation indicators of six categories.

1 $$R=\frac{2k}{1+\left(2-1\right)k}$$

where $R$ is the coding reliability and $k$ is the mutual consent degree of different encoders. According to this *Holsti* formula, $k$ is 0.833 and $R$ is 0.909.

### Instrument

To improve the validity of the scale and increase the comparability of research results, this study invited five experts to evaluate the degree of match between the measurement indicators and the concept of the relative acquisition and further simplified the scale. Subsequently, we sent 26 questionnaires to survey subjects to ask about the expression of measuring items. Finally, a formal scale with 17 indicators was formed, which is shown in Table 1.

**Table 1:** Measurement dimensions and manifestations of relative acquisition of HPAP

Variable	Code	Initial measurement item	Value form	Mean	SD
Economic conditions	EC1	Per capita annual household income	D	1.80	0.645
	EC2	Per capita annual household expenditure	D	1.87	0.630
	EC3	What do you think of your family's financial situation compared to other families	D	1.82	0.698
Health care	HC1	Do you have the medical insurance for urban and rural residents or the new rural cooperative medical insurance	V	1.00	0
	HC2	In addition to basic medical insurance, how many other kinds of medical security have you enjoyed	D	1.58	0.721
	HC3	The actual compensation ratio of the patients’ total medical expenses for the previous year	C	2.42	0.735
	HC4	The proportion of patients’ out-of-pocket medical expenses in annual household income	C	2.07	0.852
Health ability	HA1	The patient's current state of health	D	2.40	0.740
	HA2	Does the disease affect the patient's work ability	D	2.26	0.692
	HA3	Do patients often feel depressed and anxious	D	2.47	0.674
Social support	SS1	Did you get help from social organizations after illness	D	1.94	0.546
	SS2	Did you get help from relatives and friends after illness	D	1.96	0.538
Equity treatment opportunity	ET1	Has there been a situation after illness where you should have seen a doctor but did not see a doctor	D	2.10	0.795
	ET2	Whether it is convenient to seek medical treatment	D	2.01	0.805
	ET3	Are you satisfied with the attitude displayed during the visit	D	2.42	0.630
Health education	HE1	Does your community often conduct health education	D	1.58	0.563
	HE2	Will you take the initiative to learn health knowledge	D	1.67	0.637

In the variable type, “C” means continuous variable, “D” means virtual qualitative variable, and “V” means virtual binary variable

### Data collection

The data collection of this study mainly consists of two stages:

The first stage aims to verify the reliability and validity of the scale. In the pre-survey stage, we used Tongdao County in Hunan Province as a sample area. Considering the differences in identification criteria among regions, the selection of survey subjects in this study is determined by each region itself, which may also include low-income families and their members, children in distress, etc. According to the list provided by the Poverty Alleviation Office in Tongdao County, combined with geographical location, the study randomly selected the respondents and conducted the household survey after obtaining their consent. A total of 200 questionnaires were distributed, and 168 valid questionnaires were recovered, accounting for 84%. The questionnaire mainly has two parts: first, the basic information of HPAP objects, such as age, education level and so on; second, the evaluation of economic conditions, health care and other six aspects.

**Fig. 2:**
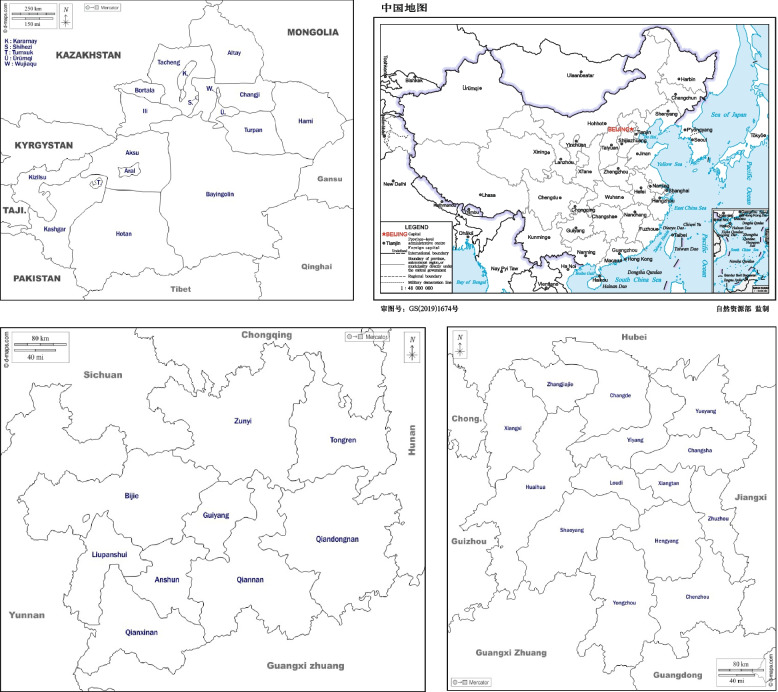
The geographic location of Qitai, Ceheng and Tongdao County

In the second stage, the study further increased the number of sample areas to ensure the scientificity of the measurement results, and the research scope was further expanded to Ceheng County in Guizhou Province and Qitai County in Xinjiang Uygur Autonomous Region which are prone to poverty and in different geographical locations (as shown in Fig. 2). The selection rules of survey subjects were the same as in the first stage. At this stage, we further distributed 360 and 200 questionnaires in Ceheng County and Qitai County with effective recoveries of 92.5% and 93%, respectively.

### Reliability and validity test

According to the requirement for the eigenvalue greater than 1, this study uses SPSS 26.0 to carry out exploratory factor analysis on the items of the scale. The result shows that the KMO value is 0.655 and passes Bartlett's test of sphericity (*P* < 0.001). According to the standard of factor loading cut-off point of 0.50, the study deleted the item with more than two factor loadings lower than or greater than 0.50, so HC1 was deleted. We used the remaining 16 items for factor analysis again and extracted 6 common factors (as shown in Table 2).

**Table 2:** Exploratory factor analysis and reliability evaluation results

Item	Economic conditions	Health care	Health ability	Social support	Equal treatment opportunity	Health education	CITC	Cronbach’s α
EC1	0.863	-0.128	0.065	0.093	0.081	-0.064	0.553	0.638
EC2	0.802	0.160	0.116	-0.113	-0.077	-0.036	0.497	
EC3	0.808	0.007	-0.031	0.161	0.053	0.046	0.514	
HC1		-a						0.632
HC2	0.126	0.575	-0.239	-0.135	0.177	0.127	0.410	
HC3	0.066	0.769	0.084	-0.032	-0.058	-0.145	0.454	
HC4	-0.159	0.775	-0.217	0.168	0.012	0.037	0.482	
HA1	0.000	-0.245	0.806	0.216	-0.007	0.072	0.830	0.922
HA2	0.152	-0.043	0.850	-0.010	-0.014	0.145	0.833	
HA3	0.003	-0.032	0.813	0.136	0.074	-0.101	0.851	
SS1	-0.015	0.085	0.166	0.841	0.000	0.042	0.560	0.703
SS2	0.156	-0.082	0.110	0.849	0.044	-0.033	0.560	
ET1	0.036	-0.001	0.013	-0.078	0.874	0.010	0.732	0.867
ET2	0.074	0.042	-0.044	0.079	0.888	0.091	0.766	
ET3	-0.049	0.049	0.077	0.050	0.840	-0.013	0.757	
HE1	-0.056	-0.009	0.056	-0.033	0.007	0.836	0.554	0.706
HE2	0.015	-0.011	0.020	0.042	0.045	0.837	0.554	

“- α” means that more than two factor loads of this item were lower than or greater than 0.50, so it was deleted

Then, the study further carried out reliability analysis on the six factors and their observed variables. The reliability evaluation results shown in Table 2 suggest that the total correlation of corrected items (CITC) of the remaining items is between 0.41 and 0.85, all above the 0.3 acceptable standard. Cronbach's a is between 0.63 and 0.92, and Cronbach's a of the overall scale is 0.664, which is greater than the 0.6 acceptable standard. The reliability of the scale is good.

Furthermore, the study used AMOS 23.0 for confirmatory factor analysis to test the structural validity, convergent validity and discriminant validity of the scale. The results show that X^2^/df = 1.485, RMSEA = 0.040, CFI = 0.947, NFI = 0.961 and IFI = 0.950, which indicates that the fitting degree between the data and scale is good and the scale has a high structural validity.

Table 3 shows that the standardized factor loading coefficients of six factors are more than 0.50 (*P* < 0.001), which indicates that the relative acquisition of HPAP can be explained by six first-order factors. The average variance extracted (AVE) of each factor is greater than 0.5, and the composite reliability (CR) value is close to or greater than 0.7, indicating that the scale has high convergent validity.

**Table 3:** Standardized factor load, AVE and CR values of CFA of relative acquisition of HPAP

Measurement model path	Standardized factor load	AVE	CR
EC3 <--- Economic conditions	0.651	0.554	0.782
EC2 <--- Economic conditions	0.613		
EC1 <--- Economic conditions	0.930		
HC4 <--- Health care	0.881	0.501	0.740
HC3 <--- Health care	0.699		
HC2 <--- Health care	0.485		
HA3 <--- Health ability	0.693	0.580	0.804
HA2 <--- Health ability	0.719		
HA1 <--- Health ability	0.863		
SS2 <--- Social support	0.775	0.535	0.696
SS1 <--- Social support	0.686		
ET3 <--- Equal treatment opportunity	0.720	0.643	0.834
ET2 <--- Equal treatment opportunity	0.883		
ET1 <--- Equal treatment opportunity	0.795		
HE2 <--- Health education	0.724	0.502	0.669
HE1 <--- Health education	0.694		

Finally, based on the study of Chin WW [27], we further tested the discriminant validity of the evaluation scale. The exploratory factor analysis results in Table 2 demonstrate that the loading of the six dimensions on the principal component is significantly greater than the cross loading on other components, indicating that the scale has good discriminant validity.

### Fuzzy comprehensive evaluation

Since there are many subjective indicators in the evaluation scale, which are difficult to measure accurately, we chose the fuzzy comprehensive evaluation method.

#### Set measure function

Firstly, the fuzzy set representing the relative acquisition of HPAP should be established and the membership function should be calculated. Specifically, if fuzzy set $X$ represents the state of relative acquisition and $Z$ is its subset, then the function of the relative acquisition of HPAP of the nth object can be expressed as $Z\left(n\right)=\left\{x,{\mu }_{Z}\left(x\right)\right\}$, where $x\in X$, ${\mu }_{Z}\left(x\right)$ means the membership function of $X$ with respect to $Z$, and the value at which ${\mu }_{Z}\left(x\right)$ maps each element of $X$ between 0 and 1 is the membership degree. In addition, each indicator and functional activity representing the relative acquisition has a membership degree, with values ranging from 0 to 1. Taking 0 means the worst situation and taking 1 means the best situation. When the membership value is between 0.4 and 0.6, the comprehensive evaluation value is in the medium level range [28].

#### Select membership function

It can be seen from Table 1 that the indicators selected in this study include continuous variables, virtual qualitative variables and virtual binary variables, so we use fuzzy distribution as the membership function. We assumed that ${x}_{i}$ represents the ith functional activity set of the relative acquisition of HPAP, and ${x}_{ij}$ represents the jth indicator of the ith functional activity set. Whether the indicator is continuous or virtual qualitative, as long as the direction of change in the value is consistent with the positive direction of change in the relative acquisition of HPAP, then we used the S- distribution to represent the membership function, as shown in Formula (2):

2 $$\mu_Z\left(x_{ij}\right)=\left\{\begin{array}{ccc}0,&x_{ij}<a\\\frac{x_{ij}-a}{b-a},&{a\leq x}_{ij}\leq b\\1,&x_{ij}>b\end{array}\right.$$

Among them, $a={x}_{ij}^{min}$, $b={x}_{ij}^{max}$. For continuous index ${x}_{ij}$, if the value of $a$ is less than the minimum value ${x}_{ij}^{min}$, meaning that its condition under this index must be poor, then ${\mu }_{Z}\left({x}_{ij}\right)=0$; if the value of $a$ is greater than the maximum value ${x}_{ij}^{max}$, then ${\mu }_{Z}\left({x}_{ij}\right)=1$. In contrast, if the direction of change in the indicator value of a continuous or virtual qualitative variable is consistent with the reverse of the direction of change in the relative acquisition of HPAP, then we used the Z-distribution to represent the membership function, as shown in Formula (3):

3 $$\mu_Z\left(x_{ij}\right)=\left\{\begin{array}{ccc}1,&x_{ij}<a\\\frac{x_{ij}-a}{b-a},&{a\leq x}_{ij}\leq b\\0,&x_{ij}>b\end{array}\right.$$

Here, $a={x}_{ij}^{min}$, $b={x}_{ij}^{max}$. For continuous index ${x}_{ij}$, if the value of $a$ is less than the minimum value ${x}_{ij}^{min}$, meaning that its condition under this index must be great, then ${\mu }_{Z}\left({x}_{ij}\right)=1$; if the value of $a$ is greater than the maximum value ${x}_{ij}^{max}$, then ${\mu }_{Z}\left({x}_{ij}\right)=0$.

Because the value of the virtual binary variable is either one or the other, the membership function is expressed as follows in Formula (4):

4 $${\mu }_{Z}\left({x}_{ij}\right)=\left\{\begin{array}{c}1,{x}_{ij}="Yes"\\ 0,{x}_{ij}="No"\end{array}\right.$$

#### Determine index weight

Because not all indicators are of equal importance to the evaluation, reasonable setting of index weight is a crucial step in the comprehensive evaluation method. This study refers to the weight setting method of the welfare level measurement index by Gao JY et al. [29] and takes $f\left(x\right)={x}^{a}(a\in \left(-\mathrm{1,0}\right))$ as the weight function. Considering the characteristics of the marginal decrease of the weight value with the increase of the index value and the monotonically change of the welfare level when the index value changes, we set a =—0.5. Then, the weight function of the jth indicator in the ith functional activity can be expressed as Formula (5):

5 $${w}_{ij}={\left\{{\mu }_{Z}({x}_{ij})\right\}}^{\left(-0.5\right)}$$

#### Add up indicators

This paper adopts the weight aggregation method proposed by Cerioli A et al. [30] to synthesize the values of the membership degree of specific indicators. Then, the calculation process of the ith functional activity value can be expressed as Formula (6). In addition, $m$ represents the number of indicators included in the ith functional activity.

6 $$Z\left(x_i\right)={\textstyle\sum\nolimits_{j=1}^m}\mu_Z(x_{ij})\times w_{ij}/{\textstyle\sum\nolimits_{j=1}^m}w_{ij}$$

## Results

### Descriptive statistic

IN the pre-survey stage, the result of questionnaires reveals that the male proportion is 53.57% and the female proportion is 46.43%; in terms of age structure, the samples aged 20 and below, 21 to 40, 41 to 60, 61 to 80, and 81 and above accounted for 1.79%, 7.14%, 41.07%, 39.29% and 10.71%, respectively; in terms of education level, the samples with primary school education or below, middle or high school education, and university education or above are 73.21%, 21.43% and 5.36%, respectively, and more than 50% of those with primary school education and below had no educational experience.

Including the valid questionnaires from Tongdao County, 687 questionnaires were collected in total in the formal survey stage. In terms of gender, 47.16% of the interviewees were male and 52.84% were female, with a relatively balanced proportion. In terms of age structure, interviewees were mainly between 41–80 years old. Among them, 49.78% were 41–60 years old, and 25.33% were 61–80 years old. In terms of education level, 71.18% of the respondents had a primary school education or below, 22.27% had a middle or high school education, and 2.18% had a university education or above. The education level of the interviewees was generally low. Specific sample characteristics are shown in Table 4.

**Table 4:** Descriptive analysis of the main characteristics of the sample

Item	category	Tongdao County		Ceheng County		Qitai County		Total	
		Number	Proportion	Number	Proportion	Number	Proportion	Number	Proportion
Age	20 years old and below	3	1.79	0	0.00	3	1.61	6	0.87
	21 to 40 years old	12	7.14	87	26.13	18	9.68	117	17.03
	41 to 60 years old	69	41.07	159	47.75	114	61.29	342	49.78
	61 to 80 years old	66	39.29	60	18.02	48	25.81	174	25.33
	81 years old and above	18	10.71	27	8.11	3	1.61	48	6.99
Gender	Male	90	53.57	147	44.14	87	46.77	324	47.16
	Female	78	46.43	186	55.86	99	53.23	363	52.84
Education	Primary school education or below	123	73.21	282	84.68	72	38.71	489	71.18
	Middle or high school education	36	21.43	36	10.81	84	45.16	153	22.27
	University education or above	9	5.36	15	4.50	0	0.00	15	2.18
	Total	168	168	100.00	333	100.00	186	100.00	687

### Fuzzy comprehensive evaluation results

The results of the fuzzy comprehensive evaluation are shown in Table 5. For 2020, the evaluation value of the relative acquisition of HPAP in survey areas is 0.4913, which is in the middle range of 0.4–0.6. Specifically, the evaluation values of each survey area are all above 0.4, of which the values of Tongdao County and Qitai County are 0.4926 and 0.4948, respectively, both greater than 0.4913, while the value of Ceheng County is only 0.4474.

**Table 5:** Evaluation value of functional activities and indicators of relative acquisition of HPAP

Item	2020		Tongdao County		Ceheng County		Qitai County	
	Membership	Weight	Membership	Weight	Membership	Weight	Membership	Weight
R	0.4913	/	0.4926	/	0.4474	/	0.4948	/
EC	0.3834	1.6150	0.5064	1.4053	0.3610	1.6644	0.2328	2.0726
EC1	0.3566	1.6745	0.4762	1.4491	0.3571	1.6733	0.1774	2.3741
EC2	0.3770	1.6287	0.5655	1.3298	0.3512	1.6874	0.1505	2.5774
EC3	0.4192	1.5445	0.4821	1.4402	0.3750	1.6330	0.4677	1.4622
HC	0.5473	1.3517	0.6649	1.2264	0.3074	1.8035	0.5993	1.2917
HC2	0.3319	1.7358	0.5625	1.3333	0.2414	1.1067	0.5806	1.3123
HC3	0.5495	1.3490	0.5921	1.2996	0.5320	1.3710	0.6603	1.2307
HC4	0.8991	1.0546	0.8818	1.0649	0.8321	1.0963	0.5615	1.3345
HA	0.6804	1.2124	0.5885	1.3036	0.7579	1.1487	0.6119	1.2784
HA1	0.6834	1.2097	0.5714	1.3229	0.8125	1.1094	0.5645	1.3310
HA2	0.6135	1.2767	0.5625	1.3333	0.7143	1.1832	0.5081	1.4029
HA3	0.7511	1.1539	0.6339	1.2560	0.7500	1.1547	0.7984	1.1192
SS	0.4857	1.4348	0.4098	1.5620	0.4999	1.4143	0.5322	1.3708
SS1	0.3553	1.9461	0.3839	1.6139	0.4911	1.4270	0.5403	1.3604
SS2	0.4934	1.4236	0.4375	1.5119	0.5089	1.4018	0.5242	1.3812
ET	0.5948	1.2967	0.6218	1.2681	0.5610	1.3351	0.6732	1.2188
ET1	0.5349	1.3673	0.6339	1.2560	0.5536	1.3440	0.4839	1.4376
ET2	0.5328	1.3701	0.5446	1.3550	0.4464	1.4967	0.7177	1.1804
ET3	0.7380	1.1641	0.6964	1.1983	0.7143	1.1832	0.8790	1.0666
HE	0.3360	1.7252	0.2845	1.8750	0.3392	1.7171	0.4875	1.4322
HE1	0.3231	1.7591	0.2589	1.9652	0.3304	1.7398	0.4677	1.4622
HE2	0.3493	1.6919	0.3125	1.7889	0.3482	1.6946	0.5081	1.4029

Furthermore, among the specific functional activities of the relative acquisition of HPAP, the values of "health care", "health ability" and "equal treatment opportunity" are higher than the average level of 0.4913, standing at 0.5473, 0.6804 and 0.5948, respectively. The values of "economic conditions", "social support" and "health education" are lower than the average level standing at 0.3834, 0.4857 and 0.336, respectively. Of course, a high value for functional activities does not mean that the value of their indicators is also high, and there are still wide differences between regions. Taking the dimension of "health care" as an example, the values of Tongdao County and Qitai County are 0.6649 and 0.5993, respectively, while Ceheng County is only 0.3074, which is significantly lower than that of the other two regions.

### Extended analysis results

Affected by personal, environmental and other factors, the process and efficiency of HPAP are different in different regions. Sen called this kind of variable, which represents the individual characteristics of HPAP, conversion factors [31]. Based on the survey data, this study selected gender, education level and type of medical insurance as conversion factors to analyze further the relative acquisition of HPAP. As shown in Table 6, from the perspective of gender, the relative acquisition of males and females are 0.4905 and 0.486, respectively, with the value for males being slightly higher than that for females, especially in the functional activities of "economic conditions" and "equal treatment opportunity"; in terms of education level, the values for the relative acquisition of the primary school or below, middle or high school education, and university education or above demographics were 0.4537, 0.5269 and 0.5891, respectively. In regard to "economic conditions" and "health ability", the value for the university education or above demographic is significantly higher than that of the other two categories.

**Table 6:** Extended analysis of the relative acquisition of HPAP under different conversion factors

Item	Gender		Education			Medical insurance			
	Male	Female	Primary school education or below	Middle or high school education	University education or above	①	① + ②	① + ③	① + ② + ③
R	0.4905	0.4860	0.4537	0.5269	0.5891	0.3688	0.4869	0.4825	0.5226
EC	0.4088	0.3676	0.3377	0.3040	0.6644	0.3839	0.5404	0.2985	0.4574
EC1	0.3826	0.3404	0.3106	0.2680	0.6667	0.3864	0.4545	0.2222	0.4286
EC2	0.3977	0.3641	0.3409	0.2222	0.7333	0.4091	0.6364	0.2484	0.5000
EC3	0.4489	0.4007	0.3636	0.4706	0.6000	0.3580	0.5455	0.4804	0.4464
HC	0.5795	0.5239	0.4419	0.5754	0.5874	0.1153	0.6399	0.5598	0.7946
HC2	0.4034	0.2872	0.1818	0.4804	0.0000	0.0057	0.5909	0.5000	0.9286
HC3	0.5541	0.5466	0.5387	0.6149	0.5125	0.5651	0.5960	0.5532	0.6526
HC4	0.8698	0.9174	0.8938	0.6451	0.6731	0.9504	0.7440	0.6344	0.8281
HA	0.6375	0.7066	0.7020	0.6688	0.9322	0.7633	0.6808	0.5982	0.5870
HA1	0.6250	0.7199	0.7045	0.6765	1.0000	0.8182	0.6818	0.5784	0.5357
HA2	0.5568	0.6489	0.6307	0.5784	0.9000	0.6932	0.7273	0.4902	0.5714
HA3	0.7443	0.7553	0.7784	0.7647	0.9000	0.7841	0.6364	0.7549	0.6607
SS	0.4730	0.4928	0.4857	0.5537	0.5916	0.4857	0.4545	0.5098	0.4103
SS1	0.4375	0.5035	0.4773	0.5686	0.7000	0.4773	0.4545	0.5098	0.4286
SS2	0.5114	0.4823	0.4943	0.5392	0.5000	0.4943	0.4545	0.5098	0.3929
ET	0.6165	0.5811	0.5391	0.6851	0.6649	0.5065	0.6281	0.6352	0.6205
ET1	0.5682	0.5142	0.4830	0.5294	0.6000	0.4830	0.6818	0.5098	0.5893
ET2	0.5455	0.5248	0.4602	0.6961	0.7000	0.3977	0.5000	0.6176	0.5536
ET3	0.7557	0.7270	0.7045	0.8725	0.7000	0.6761	0.7273	0.8137	0.7321
HE	0.3169	0.3475	0.3180	0.4847	0.3464	0.3177	0.2033	0.3911	0.3746
HE1	0.2898	0.3440	0.3068	0.4608	0.4000	0.3011	0.1818	0.3627	0.3929
HE2	0.3466	0.3511	0.3295	0.5098	0.3000	0.3352	0.2273	0.4216	0.3571

①represents basic medical insurance; ②represents critical illness insurance; ③represents medical assistance

Furthermore, in terms of the type of medical insurance, the relative acquisition of subjects who received basic medical insurance, critical illness insurance and medical assistance simultaneously had the highest value of relative acquisition of 0.5226, while the subjects who only received basic medical insurance had the lowest value of 0.3688. If the subject is covered by critical illness insurance or medical assistance, its value increases to 0.4869 and 0.4825.

## Discussion

Under the framework of the capability approach, this study defined the concept, established a theoretical model, and developed an evaluation scale for the relative acquisition of HPAP. Then, we used the fuzzy comprehensive evaluation method to measure the evaluation value of the relative acquisition of HPAP. Considering the conversion factors such as gender, education level and type of medical insurance, this paper makes a further extended analysis.

First, the relative acquisition in the dimension of "economic conditions" is slightly low, especially in the indicator of “family income”, which verifies that disease can affect the income ability of patients and their families, and this impact is usually long-term [32]. Affected by economic development level, transportation and other factors, the "economic conditions" of HPAP objects are obviously different among regions. For example, the relative acquisition of Tongdao County in the central region is 0.5064, while the values of Ceheng County and Qitai County in the southwest and northwest regions are 0.361 and 0.2328, respectively, which are significantly lower than the medium level of 0.4.

However, the study found that low-income families may also have a higher relative acquisition. Taking Qitai County as an example, the indicator value of “annual income” is only 0.1774, but in the comparison with other families, the relative acquisition value reaches 0.4677. Through further research, we find that compared with their own past status, the people in the study may pay more attention to comparison with other individuals in social relations. Enjoying higher medical compensation and more medical resources make them feel that their social status has been greatly improved, and their sense of relative acquisition has been greatly improved.

Second, the equity of HPAP has been continuously improved, especially in the dimension of "equal treatment opportunity". The interviewees said that after the implementation of the HPAP, they were no longer worried about delaying treatment for economic reasons, and they could make an appointment with an ideal doctor without the help of acquaintances. In addition, combined with the indicator of the "medical insurance" dimension in Table 6, we found that the values of “health ability” are relatively low for patients who enjoy more types of medical insurance. To some extent, this finding reflects that the current HPAP has strong pertinence [33]. Patients with high medical demands can generally enjoy more medical insurance, which significantly improves the overall health ability of HPAP objects.

Third, "social support" and "health education" have become important factors restricting the improvement of the relative acquisition of HPAP at the present stage. Through the survey, we found that the interviewees generally expressed that the help from social organizations was significantly less than that from relatives and friends. Why? On the one hand, this fact is directly related to the long-standing kinship and blood relations in rural areas of China [34]. On the other hand, it also shows that current Chinese society, especially in remote rural areas, has not established an effective social support network, and the utilization of social capital is limited. This is certainly related to the relative lag in economic development and education. In addition, the lack of health knowledge publicity by relevant departments is also a very important reason, which will reduce the output effect of the government's input through HPAP to a certain extent.

Fourth, we found that conversion factors also have an impact on the relative acquisition. For example, from the perspective of gender, the value of males is slightly higher than that of females. Because men are usually the main labour force in the family in sample areas, and their health status has a great impact on the family’s economic condition. Therefore, they generally have "priority qualification" for medical treatment at home. In regard to functional activities "health ability" and "social support", women's value is slightly higher than men because women are generally less engaged in heavy physical labour. Moreover, in Chinese rural areas, women are an important link in communication between the family and relatives. In terms of education level, objects with high education levels usually have higher relative acquisition. In the face of diseases, these people tend to adopt more scientific coping measures and have a stronger ability to eliminate negative emotions such as depression and irritability [35, 36].

## Conclusion

From the perspective of HPAP objects, this study constructs a scale and uses the fuzzy comprehensive method to evaluate the relative acquisition and equity of China's HPAP. The results show that the relative acquisition in survey areas has reached the medium level range of 0.4–0.6, and the highest level in Qitai County has reached 0.4948. Among specific functional activities, with the exception of the values of "economic conditions" and "health education," which are lower than the medium level range (0.4), values are all in the medium level range and above, which means that HPAP plays an important role in improving health status and equity. However, it should be noted, the HPAP objects generally have strong material dependence and a lack of endogenous motivation, which is not conducive to achieving long-term poverty alleviation.

In view of these problems, we should speed up the establishment of long-term mechanisms to prevent and resolve poverty caused by disease. Especially under the combined influence of multiple factors, such as ageing of population, the change of disease spectrum, the risk of chronic diseases will exist for a long time. Improving the multilevel medical security system, especially supplementary medical insurance, is of great significance for meeting the health needs of patients at different levels and preventing illness from causing poverty. At the same time, we should also strengthen the connection between different levels of the medical security system and improve the governance ability of medical insurance and the efficiency of funds.

In the long run, to improve the relative acquisition of HPAP targets and all insured people, it is necessary to achieve substantial freedom by improving various functional activities, including "economic conditions", "health ability" and "social support". International experience also shows that when poverty alleviation enters a difficult stage, stimulation by internal forces is more effective than external assistance [37, 38]. In this context, improving the level of health education is a key aspect. It is also helpful for increasing the equity of HPAP.

Based on the implementation background of China's HPAP, we studied the related problem of the relative acquisition and equity of HPAP. The data mainly come from different provinces in China. However, due to China's vast territory, there are differences in economic development level, cultural customs and other aspects among provinces, cities and regions, and there are also differences in the measures taken by HPAP in various regions. The HPAP objects themselves are heterogeneous, which makes it difficult to ensure that the research conclusions of this study are completely consistent with the situations of other regions. In this regard, we will improve results further by obtaining more detailed data and carrying out tracking research in the future.

## Data Availability

The datasets generated and analyzed during the current study are not public as they are being used by our research team in another study, but are available from the corresponding author on reasonable request.

## Abbreviations

HPAP: Health poverty alleviation project
VA: Vertical acquisition
HA: Horizontal acquisition
CITC: Total correlation of corrected items
AVE: Average variance extracted
CR: Composite reliability
